# Post-lung transplant outcomes of connective tissue disease-related interstitial lung diseases compared with idiopathic interstitial pneumonia: a single-center experience in Japan

**DOI:** 10.1007/s11748-024-02073-3

**Published:** 2024-09-06

**Authors:** Miho Yamaguchi, Takafumi Yamaya, Mitsuaki Kawashima, Chihiro Konoeda, Hidenori Kage, Masaaki Sato

**Affiliations:** 1 Department of Thoracic Surgery, The University of Tokyo Graduate School of Medicine, Tokyo, Japan; 2 Department of Respiratory Medicine, The University of Tokyo Graduate School of Medicine, Tokyo, Japan

**Keywords:** Lung transplantation, Connective tissue disease-related interstitial lung disease, CTD-ILD, Interstitial lung disease

## Abstract

**Objectives:**

The aim of this study was to investigate the outcomes of lung transplantation for connective tissue disease-related interstitial lung disease (CTD-ILD) conducted at our institution, compared with those for idiopathic interstitial pneumonias (IIPs).

**Methods:**

We retrospectively reviewed patients with CTD-ILD and IIPs who underwent lung transplantation at our hospital from July 2015 to October 2023. We compared patients’ backgrounds, early complications within 28 days post-transplant (CTCAE grade 3 or higher), postoperative courses, and prognoses between the two groups.

**Results:**

The CTD-ILD group (*n* = 19) and the IIPs group (*n* = 56) were compared. The CTD-ILD group had significantly higher preoperative use of corticosteroids and antifibrotic agents, mean pulmonary arterial pressure, anti-human leukocyte antigen antibody positivity, and donor age (*p* < 0.05). In addition, the CTD-ILD group had significantly longer operation times (579.0 vs 442.5 min), longer stays in the intensive care unit (17.0 vs 9.0 days) and hospital (58.0 vs 44.0 days); required more tracheostomies (57.9 vs 25.0%); and experienced more respiratory (52.6 vs 25.0%) and gastrointestinal (42.1 vs 8.9%) complications (*p* < 0.05). However, there were no significant differences in overall survival, nor chronic lung allograft dysfunction (CLAD)-free survival between the two groups.

**Conclusion:**

Perioperative complications, notably respiratory and gastrointestinal complications, were prevalent after lung transplantation among CTD-ILD patients. Despite this, long-term survival rates were comparable to those observed in IIP cases.

## Key points

- This study retrospectively examines lung transplantation in patients with CTD-ILD. Some centers refrained from lung transplantation because of concerns about postoperative complications and prognosis.
- There were more acute respiratory and gastrointestinal complications after lung transplantation in CTD-ILD patients compared with the IIPs group.
- The post-transplant prognosis after lung transplantation in the CTD-ILD group was similar to that of the IIPs group.

## Introduction

Interstitial lung disease (ILD) is the most predominant primary disease among lung transplant recipients, constituting 31.8% of cases, according to international registry data [1]. In Japan, ILD accounts for approximately 55% of all deceased donor lung transplantations and 34% of living-donor lung transplants [2]. However, among different types of interstitial pneumonia indicated for lung transplantation, connective tissue disease (CTD) constitutes a relatively small percentage, reportedly only 0.9% of adult lung transplantation cases in the 2019 International Society for Heart and Lung Transplantation (ISHLT) registry database [1]. Data specific to lung transplantation in Japan remain limited. According to a single-center study reported from Kyoto University, CTD patients accounted for 30.7% of patients on the lung transplant waiting list [3], which may be higher than that overseas, but the percentage of patients who actually underwent lung transplantation was not reported. The indication for lung transplantation for connective tissue disease-related interstitial lung disease (CTD-ILD) has been subject to debate because of the extrapulmonary complications associated with CTD and the uncertain prognosis. For example, patients with systemic sclerosis (SSc) often exhibit esophageal involvement, and the associated gastroesophageal reflux is a recognized risk factor for acute fatal pneumonia and chronic lung allograft dysfunction (CLAD) [4]. As a result, certain lung transplant centers have been hesitant to perform lung transplantation for SSc.

However, comparing lung transplantation for SSc with other ILDs showed no difference in the incidence of CLAD, despite the presence of severe esophageal dysmotility and gastroesophageal reflux disease (GERD) in SSc patients [5–9]. These reports also identified female sex [9], severe pulmonary hypertension (PH), and a high body mass index (BMI) [7, 9] as poor prognostic factors in lung transplantation for SSc, emphasizing the importance of patient selection within this population. In addition, a recent review article suggested that both short-term and long-term survival after lung transplantation in CTD-ILD patients may be comparable to patients with other ILDs, such as idiopathic pulmonary fibrosis (IPF), with no increased incidence of complications post-transplant [10]. While the current consensus advocates an acceptable approach to lung transplantation in selected CTD-ILD patients, there remains a scarcity of reports specifically addressing CTD-ILD, particularly in Asia. Therefore, we investigated the post-transplant complications and prognosis of patients with CTD-ILD who underwent lung transplantation at our hospital.

## Patients and methods

### Population

This research constitutes a retrospective, descriptive, and exploratory analysis of an ongoing cohort, approved by the Ethics Committees of the University of Tokyo Hospital [IRB#: 2406-(9)]. The study included all adult recipients who underwent lung transplantation at our hospital between July 1st, 2015 and November 30th, 2023, with a follow-up period until December 31, 2023.

### Diagnosis of CTD-ILD and IIPs

The diagnosis of CTD-ILD or IIP was made through discussion in the lung transplant team as well as the intra-institutional committee evaluating lung transplant candidates at the time of listing a patient, considering the medical history, images, blood tests and the original clinical diagnosis made at the center referring the patient to us. In cases where new data emerged to indicate CTD-ILD in a patient already listed as IIP during the waiting time, discussion was made in the lung transplant team and the diagnosis was changed if necessary.

Among these recipients, individuals diagnosed with either CTD-ILD or IIPs at the time of lung transplant listing were identified and classified into their respective groups. In addition, patients whose diagnosis was changed from IIPs to CTD when their lungs were explanted at the time of transplantation were also included in the CTD-ILD group.

### Measures and study outcomes

Characteristics of both donors and recipients, as well as details of surgical and postoperative courses, were extracted from medical records. Donor and recipient factors were recorded at the time of transplantation, with exceptions for specific parameters such as mean pulmonary arterial pressure (mPAP), percentage of predicted forced vital capacity (%FVC), percentage of predicted diffusing capacity of the lungs for carbon monoxide (%DLCO), the 6-min walk test (6MWT), and KL-6, which were recorded at the time of listing for lung transplantation. Among donor factors, age, sex (male), BMI, and partial pressure of oxygen fraction of inspired oxygen ratio (P/F) were analyzed, with living donors excluded from the analysis. Postoperative complications were categorized according to the common terminology criteria for adverse events (CTCAE ver 5.0) of grade 3 or above, occurring within 28 days postoperatively. These complications included categories such as mental/neurological, cardiovascular, respiratory, acute rejection, gastrointestinal, renal/electrolyte, hematology and infections in extra respiratory organ. Pneumonia diagnosis was based on the guidelines outlined by the Japan Respiratory Society for the management of pneumonia in adults [11]. The diagnosis of acute rejection relied on clinical assessments, which included radiographic images, blood tests, and other clinical findings, with or without transbronchial biopsy [12–14]. Furthermore, diagnoses of CLAD and primary graft dysfunction (PGD) grade 3 occurring within 72 h were determined in accordance with the criteria defined by the International Society for Heart and Lung Transplantation [15, 16].

### Patient management

All patients received treatment in accordance with the postoperative lung transplant protocol of the University of Tokyo Hospital. The immunosuppression regimen included calcineurin inhibitors (tacrolimus or cyclosporine), antimetabolites (mycophenolate mofetil or azathioprine), and corticosteroids (prednisolone). Tacrolimus targeted trough levels were maintained at 11–14 ng/mL until the third postoperative month, 9–13 ng/ml until the 6th month, and 8–12 ng/ml thereafter. Infection prophylaxis included cytomegalovirus prophylaxis for 6 months in non-mismatched patients and 12 months in mismatched patients, antifungal agents (primarily itraconazole) for life, and sulfamethoxazole–trimethoprim for life. Regular monitoring included laboratory pulmonary function tests, CT scans, and anti-human leucocyte antigen (HLA) antibody measurements every 3 months during the first-year post-transplantation, followed by assessments every 6 months thereafter.

### Statistics

Continuous variables are presented as median (interquartile range, IQR), and were compared using the Mann–Whitney *U* test. Categorical variables are presented as percentages and were analyzed with Fisher’s exact test. Kaplan–Meier cumulative survival curves were plotted for each group, and survival rates between groups were compared using the log-rank test. All statistical analyses were conducted using EZR (Saitama Medical Center, Jichi Medical University, Saitama, Japan), which serves as a graphical user interface for R (The R Foundation for Statistical Computing, Vienna, Austria) [17].

## Results

**Fig. 1:**
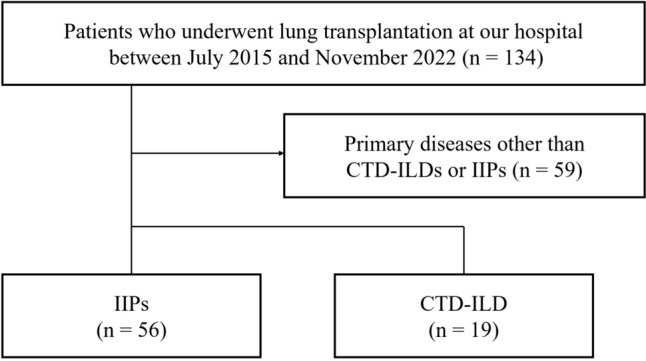
Study cohort. At our institution, the percentage of lung transplantation for CTD-ILD was 14.2% (19 out of 134 total cases), a higher frequency than previous reports (0.9% in the 2019 ISHLT registry database)

Between July 2015 and November 2023, our hospital conducted 134 lung transplants, including 19 for CTD-ILDs and 56 for IIPs (Fig. 1). No patients were excluded. The IIPs group included primary diseases such as IPF (*n* = 23), idiopathic pleuroparenchymal fibroelastosis (IPPFE, *n* = 12), nonspecific interstitial pneumonia (NSIP, *n* = 6), and others as unclassifiable (*n* = 15). Among the CTD-ILD group, background collagen diseases included SSc (*n* = 6), polymyositis/dermatomyositis (PM/DM, *n* = 5), antineutrophil cytoplasmic antibody-associated vasculitis (AAV, *n* = 3), rheumatoid arthritis (RA, *n* = 3), Sjogren’s syndrome (Sjs, *n* = 1), and mixed connective tissue disease (MCTD, *n* = 1) (Table 1). Among the patients who received lung transplantation from brain-dead donors, the CTD-ILD group had significantly longer waiting time compared with the IIP group (median, 950 days vs. 657 days (*p* = 0.012): Table 1).

**Table 1:** Patient characteristics (recipient factor, donor factor), surgical details, and postoperative course

Variable Median (IQR), *n* (% within group)	Total (*n* = 75)	IIPs (*n* = 56)		CTD-ILD (*n* = 19)		*p*
Recipient factor						
Age (years)	53.0 (44.0–58.0)	54.0 (45.8–58.3)		49.0 (39.5–57.0)		0.220
Males	47 (62.7)	39 (69.6)		8 (42.1)		0.053
BMI (kg/m^2^)	20.2 (16.5–24.2)	20.3 (16.2–24.3)		20.2 (17.2–24.0)		0.617
Smoking	43 (57.3)	33 (58.9)		10 (52.6)		0.789
Primary disease		IPF IPPFE NSIP Others	23 (41) 12 (21) 6 (11) 15 (27)	SSc PM/DM AAV RA Sjs MCTD	6 (32) 5 (26) 3 (16) 3 (16) 1 (5) 1 (5)	
Corticosteroid use	43 (57.3)	26 (46.4)		17 (89.4)		0.001
Use of immunosuppressive agents	33 (44)	21 (37.5)		12 (63.1)		0.065
Use of antifibrotic agents^*^	40 (53.3)	34 (60.7)		6 (31.6)		0.035
Dose of oxygen (L/min)	4.0 (3.0–6.0)	4.5 (3.0–7.0)		4.0 (2.5–5.3)		0.199
Diabetes	10 (13.3)	8 (14.3)		2 (10.5)		1
GERD	10 (13.3)	7 (12.5)		3 (15.8)		0.707
mPAP (mmHg)	18.5 (15.0–23.3)	17 (14.5–20)		24 (19.0–28.0)		< 0.001
%FVC (%)	49 (41.2–60.8)	49.0 (42.5–59.9)		46.6 (37.2–62.5)		0.402
%DLCO (%)	48.15 (39.0–48.2)	40.5 (31.0–49.2)		35.6 (31.0–47.3)		0.426
6MWT (m)	345 (275–432)	395 (287–450)		343 (270–413)		0.524
KL-6 (U/mL)	981.5 (557–1622)	965 (6423–1651)		1103 (425–1556)		0.553
Alb (g/dL)	3.9 (3.6–4.2)	4.0 (3.6–4.1)		3.8 (3.7–4.1)		0.706
Cre (mg/dL)	0.64 (0.48–0.80)	0.67 (0.50–0.80)		0.58 (0.38–0.78)		0.292
CRP (mg/dL)	0.45 (0.14–0.83)	0.33 (0.13–0.75)		0.65 (0.25–1.12)		0.265
LDH (U/L)	261 (212–323)	266.5 (222–329)		234 (213–310)		0.120
Donor factor						
Age (years)	53 (42.5–61.3)	51 (41.0–59.0)		59 (51.0–63.5)		0.028
Males	44 (64.7)	34 (69.4)		10 (52.6)		0.260
BMI (kg/m^2^)	23.2 (21.6–25.4)	23.2 (21.2–25.1)		23.2 (21.6–25.5)		0.769
Smoking	46 (61.3)	35 (62.5)		11 (57.9)		0.788
Sex mismatch	14 (18.7)	10 (17.9)		4 (21.1)		0.253
CMV mismatch	11 (14.7)	8 (14.3)		3 (15.8)		0.743
P/F	431 (345–509)	432 (340–512)		428 (263–642)		0.571
Surgery/postoperative course						
Waiting period (day)	757 (392–999)	657 (357–866)		950 (770–1184)		0.012
Observation period after transplant (day)	669 (319–1299)	729 (348–1336)		541 (226–880)		0.286
Living donor	6 (8.0)	6 (10.7)		0 (0)		0.328
Bilateral lung transplant	33 (44.0)	22 (39.3)		11 (57.9)		0.188
Operation time (min)	477 (366–609)	442.5 (355–564)		579.0 (481–646)		0.032
Blood loss (ml)	1555 (643–3070)	1410 (518–2656)		2620 (915–4063)		0.161
ECMO/CPB use	50 (66.7)	36 (64.3)		14 (73.7)		0.577
Mechanical ventilation (day)^†^	4.0 (3.0–7.5)	4.0 (3.0–6.0)		5.0 (4.0–16.0)		0.078
Tracheostomy	25 (33.3)	14 (25.0)		11 (57.9)		0.012
Days in ICU (day)^†^	10.0 (7.0–18.0)	9.0 (7.0–25.8)		17.0 (10.5–26.5)		0.015
Days in hospital (day)^‡^	46.0 (37.0–58.0)	44.0 (35.0–56.5)		58.0 (48.5–63.0)		0.021

*IPF* idiopathic pulmonary fibrosis, *IPPFE* idiopathic pleuroparenchymal fibroelastosis, *NSIP* nonspecific interstitial pneumonia, *SSc* systemic sclerosis, *Sjs* Sjogren’s syndrome, *RA* rheumatoid arthritis, *PM* polymyositis, *DM* dermatomyositis, *AAV* antineutrophil cytoplasmic antibody-associated vasculitis, *MCTD* mixed connective tissue disease, *mPAP* mean pulmonary arterial pressure, *6MWT* 6-min walk test, *P/F* PaO_2_/FiO_2_ ratio, *ECMO* extracorporeal membrane oxygenation, *CPB* cardiopulmonary bypass

^*^Antifibrotic agents: pirfenidone, nintedanib, or both

^†^For the duration of ventilator use and ICU stay, undischarged deaths (*n* = 3) were excluded from the analysis

^‡^For the duration of hospitalization, we excluded undischarged deaths (*n* = 3) and undischarged (*n* = 3)

Preoperative corticosteroid use was more frequent in the CTD-ILD group compared with the IIPs group, while antifibrotic drug use was significantly less frequent. The CTD-ILD group showed significantly higher mPAP (*p* < 0.001) (Table 1). Donor age was significantly higher in CTD-ILD cases, but other donor factors did not show significant differences. In addition, CTD-ILD cases experienced longer operating times (*p* = 0.032), more tracheostomies (*p* = 0.012), and longer stays in the ICU (*p* = 0.015) and hospital (*p* = 0.021) (Table 1). The CTD-ILD group underwent more bilateral lung transplantations than the IIPs group, and no patients in this group received living-donor lung transplants.

**Fig. 2:**
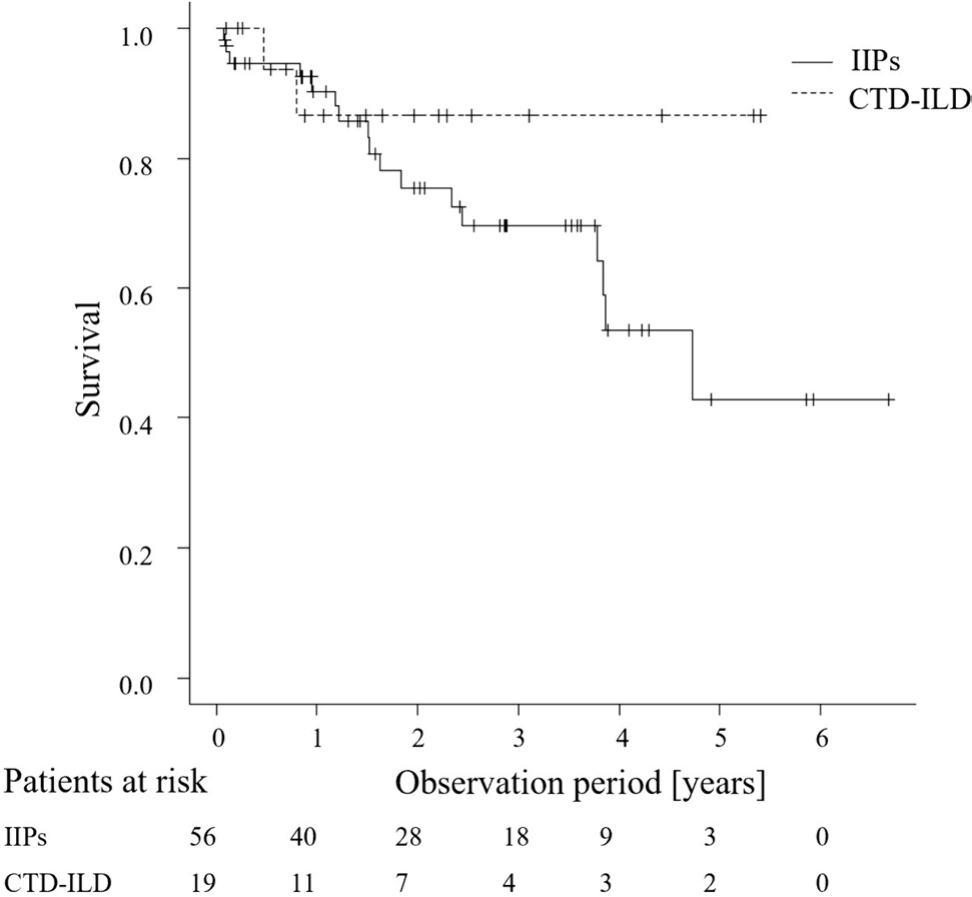
Kaplan–Meier survival curves for each group. No significant differences in post-transplant survival were observed (*p* = 0.232). The 1-year survival rate was 86.5% for CTD-ILD and 90.3% for IIPs, while the 3-year survival rate was 86.5% for CTD-ILD and 69.5% for IIPs

**Fig. 3:**
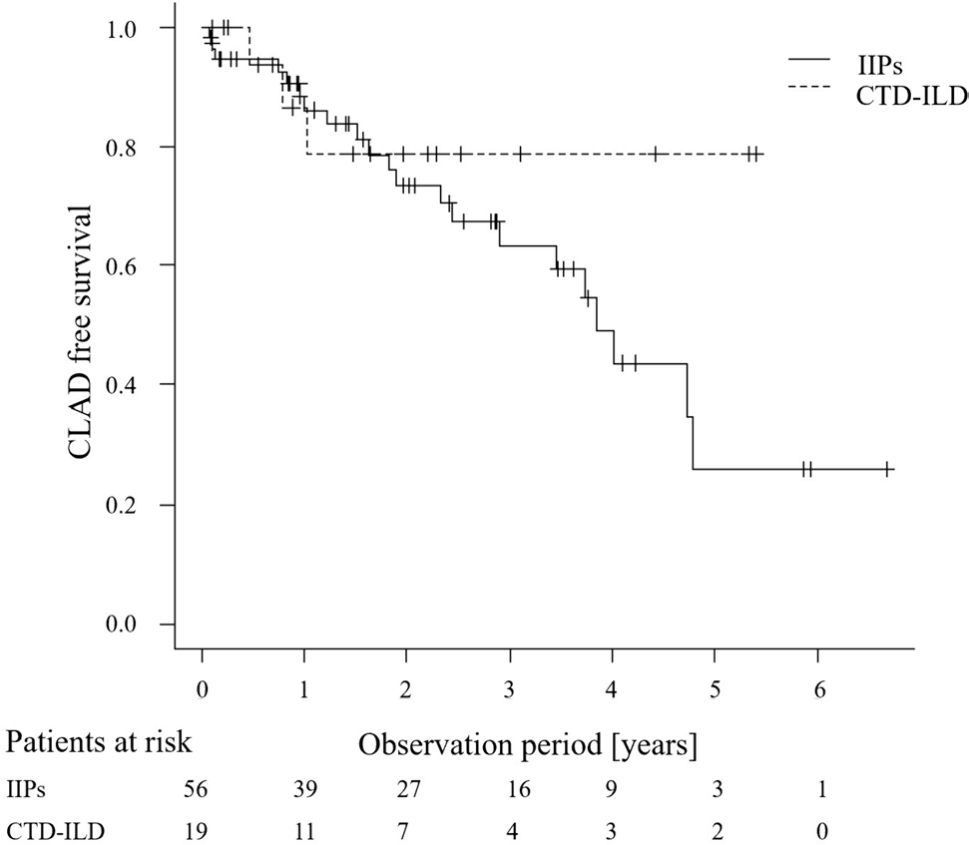
Kaplan–Meier CLAD-free survival curve was plotted for each group. No significant differences in post-transplant survival were observed (*p* = 0.239)

Pulmonary and gastrointestinal complications were significantly more common in CTD-ILDs within 28 days postoperatively (*p* = 0.0044 and *p* = 0.003, respectively; Table 2). Respiratory-related complications were primarily pneumonia and acute rejection, while gastrointestinal-related complications included constipation, diarrhea, cholecystitis, and elevated liver enzymes. Although acute rejection, kidney-related complications, and postoperative HLA antibody positivity tended to be higher in the CTD-ILD group, these differences were not significantly different (*p* = 0.326, *p* = 0.100, and *p* = 0.060, respectively). No significant differences were found in patient survival rates between the CTD-ILD and IIPs groups (Fig. 2): 1-year survival (86.5 vs 90.3%) and 3-year survival (86.5 vs 69.5%), with CTD-ILD patients tending to have better long-term survival. In addition, there was no significant difference in CLAD-free survival between the two groups (*p* = 0.239) (Fig. 3). The CTD-ILD group had a significantly higher prevalence of preformed anti-HLA antibodies (*p* = 0.048) (Table 3). However, no significant differences were observed in postoperative anti-HLA antibodies and donor-specific antibodies (DSA) (Table 3).

**Table 2:** Complications rated as CTCAE Grade 3 or higher within 28 days following lung transplantation

Variable *n* (% within group)	Total (*n* = 75)	IIPs (*n* = 56)	CTD-ILD (*n* = 19)	*p*
PGD grade 3 within 72 h	13 (17.3)	9 (16.1)	4 (21.1)	0.727
Hematoma removal procedure	11 (14.7)	8 (14.3)	3 (15.8)	1.000
Mental/neurological	11 (14.7)	8 (14.3)	3 (15.8)	1.000
Cardiovascular	8 (10.7)	5 (8.9)	3 (15.8)	0.410
Pulmonary	24 (32.0)	14 (25.0)	10 (52.6)	0.044
Acute rejection	14 (18.7)	9 (16.1)	5 (26.3)	0.326
Bacterial pneumonia	7 (9.3)	5 (8.9)	2 (10.5)	1
Others*	4 (5.3)	2 (3.6)	2 (10.5)	0.264
Gastrointestinal	13 (17.3)	5 (8.9)	8 (42.1)	0.003
Renal/electrolyte	5 (66.7)	2 (3.5)	3 (15.8)	0.100
Hematology	5 (66.7)	3 (5.4)	2 (10.5)	0.596
Infections (extra respiratory)	7 (9.3)	5 (8.9)	2 (11.8)	1.000

*PGD* primary graft dysfunction

^*^Thoracic empyema, diaphragmatic dysfunction, pneumothorax, or pleural effusion

**Table 3:** Presence of anti-HLA antibodies and donor-specific antibodies (DSA)

Variable *n* (% within group)	Total (*n* = 75)	IIPs (*n* = 56)	CTD-ILD (*n* = 19)	*p*
Preformed anti-HLA antibody	15 (20)	8 (14.3)	7 (36.8)	0.048
Preformed DSA	7 (9.3)	5 (8.9)	2 (10.5)	1
Postoperative HLA antibody*	18 (24)	10 (17.8)	8 (42.1)	0.060
Postoperative de novo HLA antibody*	10 (13.5)	7 (12.7)	3 (15.8)	0.710
Postoperative DSA*	10 (13.5)	5 (9.1)	5 (26.3)	0.112
Postoperative de novo DSA*	6 (8.1)	3 (5.5)	3 (15.8)	0.172
Postoperative de novo DSA + CREG*	9 (12.0)	6 (10.7)	3 (15.8)	0.686

*HLA* human leukocyte antigen, *DSA* donor-specific antibody, *CREG* cross reactive group

^*^One patient from the IIP group who did not undergo postoperative HLA antibody measurement died and was subsequently excluded from the analysis

During the follow-up period, there were 2 deaths in the CTD-ILD group due to antibody-mediated rejection (AMR) and multiple organ failure. In the IIPs group, there were 17 deaths: 6 from CLAD, 3 from cancer, 1 from AMR, 2 from primary graft dysfunction, 2 from multiple organ failure, 2 from infection, and 1 from an intracranial event.

## Discussion

This study compared the postoperative course of patients with CTD-ILD and IIPs who underwent lung transplantation at our hospital, with a focus on complications occurring within 28 days of surgery. Nineteen lung transplants for CTD-ILD were performed at our center out of 134 total cases (14.2%), which was higher than previous reports (0.9% in the 2019 ISHLT registry database). Significant differences were observed in recipient factors, including preoperative corticosteroid and antifibrotic drug use, mPAP, and pre-transplant anti-HLA antibody prevalence, as well as in donor age. The CTD-ILD group experienced longer operation times, more tracheostomies, longer ICU stays and hospitalization, and higher rates of respiratory and gastrointestinal complications. However, there was no significant difference in overall survival between the groups.

The risk of postoperative infection is a major concern in patients with CTDs because of preoperative immunosuppression. A previous report has indicated that recipient’s airway bacterial flora, rather than donor’s, are associated with postoperative pneumonia after lung transplantation [18], while the risk of rejection due to activation of the immune system is another significant concern. In this study, the use of corticosteroids and immunosuppressants tended to be more frequent in patients with CTD-ILD compared with IIPs, consistent with a previous report [19]. In addition, preformed anti-HLA antibodies were more commonly observed in CTD-ILD cases than in IIP cases (Tables 1 and 3). However, there was no significant difference in the incidence of acute rejection (Table 2) or the emergence of either de novo anti-HLA antibodies or de novo DSA after lung transplantation (Table 3) between the two groups. Similarly, previous reports have shown that CTD-ILD is not associated with a higher frequency of graft rejection compared with IPF [19–22]. There were also reportedly no significant differences in pulmonary infection after lung transplantation between CTD-ILD and IPF [23], which is consistent with our findings (Table 2). Conversely, a report suggested a higher probability of grade > 2 bronchiolitis obliterans syndrome in CTD-ILD compared with IPF [19], which may be due to exposure to chronic airway infection risk associated with long-term corticosteroid use. The follow-up time of the present study was not long enough to elucidate the association between CTD-ILD and the incidence of CLAD. Careful long-term follow-up is warranted.

Another significant concern in lung transplantation for CTD-ILD has been the systemic complications associated with CTDs. Systemic complications include gastrointestinal complications, renal dysfunction, and pulmonary hypertension. The presence of concomitant PH is considered an important predictor of postoperative outcomes [7, 9]. Although patients with IIPs can develop secondary PH (Group 3 PH), preoperative mPAP was significantly higher in the CTD-ILD group in this study. The elevation may be attributed to the coexistence with the Group 1 PH in this population. In the CTD-ILD group, the longer operation times are likely associated with the frequency of bilateral lung transplantation and extracorporeal membrane oxygenation use, which may also indicate underlying PH. Similarly, patients with CTD-ILD (except for SSc cases) reportedly showed higher mPAP and underwent bilateral lung transplantation more frequently than those with IPF [19].

Regarding early postoperative complications, while no differences were observed in rejection and pneumonia, as mentioned earlier, there was a significantly higher overall incidence of respiratory complications. This could have led to prolonged mechanical ventilation and a higher frequency of tracheostomies. The presence of gastrointestinal complications was also significantly higher in the CTD-ILD group than in the IPF group. Patients with CTD-ILD experienced various gastrointestinal complications such as reflux esophagitis, diarrhea, constipation, and nonspecific abdominal pain; however, in practice, such complications were not fatal. Many patients with CTD-ILD suffered from reduced gastrointestinal motility after lung transplantation. However, this condition typically improves over time with the assistance of prokinetic agents. Nonetheless, such complications may have contributed to delayed discharge from the ICU and hospital in patients with CTD-ILD.

Interestingly, we found no significant difference in survival rates between the two groups (Fig. 2). The CTD-ILD group exhibited somewhat higher survival rates at 3 years post-lung transplantation (86.5 vs. 69.5%). This finding is consistent with a previous report from Kyoto University demonstrating a relatively favorable 5-year survival rate of 86.2% after lung transplantation in SSc patients [24]. Moreover, despite concerns regarding aspiration due to gastrointestinal disorders contributing to CLAD in CTD patients, no significant difference was observed in CLAD-free survival (Fig. 3). However, these long-term outcomes need to be carefully interpreted. First, the patients’ pre-transplant conditions might be largely different between the groups. The longer waiting period in the CTD-ILD group (Table 1) and no need for living-donor transplantation indicate relatively stable pre-transplant condition of this group. Better response to immunosuppressive treatment could contribute to relatively good disease control before transplantation in the CTD group. Second, the observation period is relatively short in this study (Table 1), and thus the occurrence of CLAD and other complications (e.g., malignancy) may be underestimated. Third, the patient survival could be confounded by factors other than the underlying diagnosis. For example, the patients with CTD-ILD received bilateral rather than single lung transplantation more frequently, which could contribute to better long-term survival.

Other than the above-mentioned limitations, we acknowledge several limitations in this study. First, it is a single-center retrospective study with a limited number of patients, making multivariate analysis challenging. Second, the mixture of different entities within the CTD-ILD group may have introduced heterogeneity that could have influenced the study outcomes to an uncertain extent. Third, the relatively favorable long-term outcome of lung transplantation for CTD-ILD in this study may also be influenced by selection bias. Given the systemic nature of CTD, patient selection likely would have played a significant role in the success of lung transplantation. However, the exploration of factors used to select lung transplant candidates was beyond the scope of this study. Additional experience accumulation and prospective analysis are warranted to reveal further insights into lung transplantation for CTD-ILD.

## Conclusion

Our study compared patients with CTD-ILD to those with IIPs who underwent lung transplantation at our institution. We observed that acute complications were more prevalent in CTD-ILD patients. In lung transplantation for patients with CTD-ILD, close attention should be paid to acute management. Further investigations are needed regarding long-term prognosis.

## Data Availability

Please contact the authors directly for information on sharing the data used in this study.
