# Correction: Post-lung transplant outcomes of connective tissue disease-related interstitial lung diseases compared with idiopathic interstitial pneumonia: a single-center experience in Japan

**DOI:** 10.1007/s11748-024-02108-9

**Published:** 2024-12-02

**Authors:** Miho Yamaguchi, Takafumi Yamaya, Mitsuaki Kawashima, Chihiro Konoeda, Hidenori Kage, Masaaki Sato

**Affiliations:** 1 Department of Thoracic Surgery, The University of Tokyo Graduate School of Medicine, Tokyo, Japan; 2 Department of Respiratory Medicine, The University of Tokyo Graduate School of Medicine, Tokyo, Japan

**Correction: General Thoracic and Cardiovascular Surgery** 10.1007/s11748-024-02073-3

The sentence beginning “Between July 2015 and November 2023, …” in this article, should have read “Between July 2015 and November 2023, our hospital conducted 174 lung transplants, including 19 for CTD-ILDs and 56 for IIPs (Fig. 1).”.

The sentence beginning “Nineteen lung transplants for CTD-ILD…” under ‘Discussion’, should have read, “Nineteen lung transplants for CTD-ILD were performed at our center out of 174 total cases (10.9%), which was higher than previous reports (0.9% in the 2019 ISHLT registry database).”.

In this article, the caption to Fig. 1 should have read “Study cohort. At our institution, the percentage of lung transplantation for CTD-ILD was 10.9% (19 out of 174 total cases), a higher frequency than previous reports (0.9% in the 2019 ISHLT registry database).”

